# Correlates of neutralizing/SARS-CoV-2-S1-binding antibody response with adverse effects and immune kinetics in BNT162b2-vaccinated individuals

**DOI:** 10.1038/s41598-021-01930-y

**Published:** 2021-11-24

**Authors:** Kenji Maeda, Masayuki Amano, Yukari Uemura, Kiyoto Tsuchiya, Tomoko Matsushima, Kenta Noda, Yosuke Shimizu, Asuka Fujiwara, Yuki Takamatsu, Yasuko Ichikawa, Hidehiro Nishimura, Mari Kinoshita, Shota Matsumoto, Hiroyuki Gatanaga, Kazuhisa Yoshimura, Shin-ichi Oka, Ayako Mikami, Wataru Sugiura, Toshiyuki Sato, Tomokazu Yoshida, Shinya Shimada, Hiroaki Mitsuya

**Affiliations:** 1 Department of Refractory Viral Infections, National Center for Global Health and Medicine (NCGM) Research Institute, Tokyo, Japan; 2 Department of Clinical Sciences, Kumamoto University Hospital, Kumamoto, Japan; 3 Center of Clinical Sciences, NCGM, Tokyo, Japan; 4 AIDS Clinical Center, NCGM, Tokyo, Japan; 5 Sysmex Corporation, Hyogo, Japan; 6 JCHO Kumamoto General Hospital, Kumamoto, Japan; 7 Tokyo Metropolitan Institute for Public Health, Tokyo, Japan; 8 Experimental Retrovirology Section, HIV and AIDS Malignancy Branch, National Cancer Institute, National Institutes of Health, Bethesda, MD USA

**Keywords:** RNA vaccines, SARS-CoV-2, Viral infection

## Abstract

While mRNA vaccines against SARS-CoV-2 are exceedingly effective in preventing symptomatic infection, their immune response features remain to be clarified. In the present prospective study, 225 healthy individuals in Japan, who received two BNT162b2 doses, were enrolled. Correlates of BNT162b2-elicited SARS-CoV-2-neutralizing activity (50% neutralization titer: NT_50_; assessed using infectious virions) with various determinants were examined and the potency of sera against variants of concerns was determined. Significant rise in NT_50_s was seen in sera on day 28 post-1st dose. A moderate inverse correlation was seen between NT_50_s and ages, but no correlation seen between NT_50_s and adverse effects. NT_50_s and SARS-CoV-2-S1-binding-IgG levels on day 28 post-1st dose and pain scores following the 2nd dose were greater in women than in men. The average half-life of NT_50_s was ~ 68 days, and 23.6% (49 out of 208 individuals) failed to show detectable neutralizing activity on day 150. While sera from elite-responders (NT_50_s > 1,500: the top 4% among the participants) potently to moderately blocked all variants of concerns examined, some sera with low NT_50_s failed to block the B.1.351-beta strain. Since BNT162b2-elicited immunity against SARS-CoV-2 is short, an additional vaccine or other protective measures are needed.

## Introduction

Since the emergence of coronavirus disease 2019 (COVID-19) caused by severe acute respiratory syndrome coronavirus 2 (SARS-CoV-2) in Wuhan, China, the disease quickly spread to the world. As of June 29, 2021, more than 180 million SARS-CoV-2-infected individuals and almost 4 million death cases have been reported in over 200 countries^1–4^. Since the beginning of the pandemic, researchers and pharmaceutical companies around the world have been working on developing vaccines^5^. Currently, more than 10 vaccines have been authorized for public use worldwide. The development of vaccines against SARS-CoV-2 was achieved time- and efficacy-wise beyond our expectations within a single calendar year from the availability of the viral sequence to the initiation of immunization of many people in several countries^6,7^.

Among various vaccines, two RNA vaccines (BNT162b2 and mRNA-1273/TAK-919) have been shown to be as much as 94–95% effective and safe^8–11^. In addition, inactivated vaccines or viral vector vaccines have also been available in certain countries and areas^5,7,10,11^. For example, the adenovirus-vector-based vaccine (ChAdOx1 nCoV-19/AZD1222) has reportedly achieved 62% efficacy in initial trials^12^. The phase 3 reports of another adenovirus-based vaccine (Ad26.COV2.S) has indicated 85% efficacy against severe disease or death^13,14^.

However, the recent emergence of various SARS-CoV-2 variants with mutations in the spike region is raising concerns about the efficacy of vaccines. The D614G and B.1.1.7 (alpha/N501Y) variants appear to be without antigenic escape^15,16^. However, the B.1.351 (beta) variant is reportedly represents a neutralization escape variant to convalescent sera^17^. The phase 3 results of NVX-CoV2373 (a nanoparticle, protein-based vaccine) from the United Kingdom indicated 89% efficacy with over 50% of cases attributable to the more transmissible alpha variant^18^. However, a phase 2b trial in South Africa showed 60% efficacy, in which approximately 90% of the endpoints occurred in subjects infected with the beta variant^19,20^, suggesting that the beta variant is less susceptible to antibodies elicited with the original Wuhan strain antigens, which is in the composition of all the vaccines currently being evaluated^7^. Another recent concern is the emergence of a B.1.617.2 (delta) variant, which was first detected in India, is now spreading around the world. This variant of concern (VOC) seems to have less susceptibility to vaccine-elicited protection and increased transmissibility beyond alpha strains^21^.

In the present study, we examined neutralizing activity and S1-binding-antibody response in BNT162b2-vaccinated health care workers (n = 225) in Japan. We also investigated the correlation among neutralizing activity levels, S1-binding-IgG and -IgM levels, genders, and adverse events. Decline of BNT162b2-elicited immune response and activity of the elite and moderate responders against VOCs were also investigated.

## Results

### Demographic characteristics and immune response in the participants

We obtained blood samples for antibody testing from a total of 225, 220, 211, 210 and 208 vaccine recipients on days 7, 28, 60, 90 and 150 post-1st dose, respectively (Table 1). Demographic characteristics of the participants are shown in Table 1. As of the time of enrollment, the average age of the participants was 41.8 years (range 21–72 years), and 69.8% of the participants were female serving as a physician, nurse, paramedical staff, or administrative staff. None of the participants was in the immunodeficient state or was receiving immunosuppressants or steroids.

**Table 1:** Study protocol and demographic characteristics of the participants.

	Day7 (Post 1st dose)	(%)	Day28	Day60	Day90	Day150
All	225		220	211	210	208
**Age**						
20–39	97	43.1	92	84	84	82
40–59	110	48.9	110	109	108	109
≥ 60	18	8.0	18	18	18	17
(Average)	(41.8 y.o.)					
**Gender**						
Men	68	30.2	68	63	61	62
Women	157	69.8	152	148	149	146
**Job**						
Physicians	36	16.0				
Nurses	125	55.6				
Others	64	28.4				

**Figure 1:**
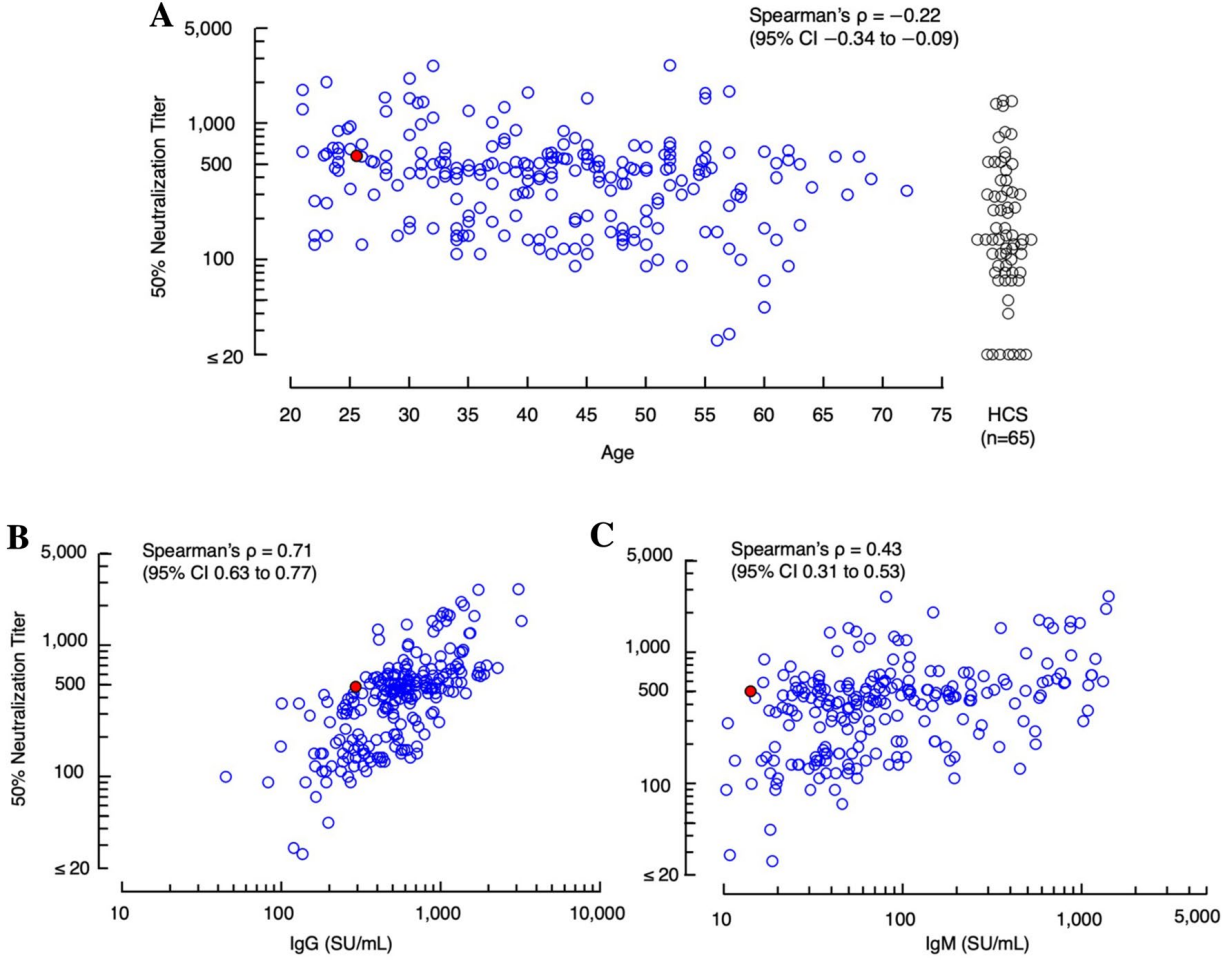
Correlations of neutralizing titers with ages and S1-binding-IgG and -IgM levels. (**A**) Correlation between neutralizing titers (NT_50_s) on day 28 post-1st dose and the ages of the participants (on day 28 post-1st dose). The age range of the study participants was 21–72 (average 41.8 y.o.). A correlation is negligible between NT_50_ values and ages (Spearman’s ρ =  − 0.22: 95% CI − 0.34 to − 0.09, *p* = 0.001). The geometric mean NT_50_ of the values from all participants (n = 225) was 375.2 (range 25.6–2680), greater by a factor of 2.3 than the geometric mean NT_50_ from 65 COVID-19-convalescent patients (geometric mean = 163.0; range 20.0–1470) shown as references on the far right (human COVID-19-convalescent serum: HCS). (**B**) A high correlation is identified (Spearman’s ρ = 0.71; 95% CI 0.63–0.77, *p* < 0.001) between NT_50_ values and S1-binding-IgG levels in samples obtained on day 28 post-1st dose. (**C**) Moderate correlation is seen between neutralizing titers and S1-binding-IgM levels (Spearman’s ρ = 0.43; 95% CI 0.31–0.53, *p* < 0.001). One participant, who had been infected with SARS-CoV-2 with PCR-positivity documented, is indicated as a solid-red solitary circle. This participant was excluded from all analyses at later timepoints.

We first determined the neutralizing activity against SARS-CoV-2 in sera samples taken on day 7 post-1st dose from 225 participants; however, none of the samples showed detectable neutralization activity (NT_50_ < 20). We then determined the neutralizing activity in samples taken on day 28 post-1st dose from 220 vaccinated participants. As shown in Fig. 1A, NT_50_ levels were substantially diverse among the participants: the geometric mean of NT_50_ values was 375.2 (range 25.6–2680.0). Very low or no correlation of the NT_50_ values with ages was identified (Fig. 1A: Spearman’s ρ =  − 0.22; 95% CI − 0.34 to − 0.09, *p* = 0.001). We also examined the levels of S1-binding-IgG and -IgM levels using the HISCL system that enables quantitative and highly sensitive determination of S1-binding-IgG and -IgM levels^22^. The geometric mean of S1-binding-IgG values was 527.0 (range 44.6–3212.2), while that of S1-binding-IgM was 85.1 (range 10.3–1406.5). There was a high positive correlation of the NT_50_ values with S1-binding-IgG levels (Spearman’s ρ = 0.71; 95% CI 0.63–0.77, *p* < 0.001) as examined on day 28 post-1st dose, while there was a moderate positive correlation of the NT_50_ values with S1-binding-IgM levels (Spearman’s ρ = 0.43; 95% CI 0.31–0.53, *p* < 0.001), suggesting that the neutralizing activity largely resides in IgG fraction of the sera of vaccinated participants around on day 28 post-1st dose (Fig. 1B, C). However, when examined on day 60 post-1st dose, the correlations of NT_50_ levels with both IgG and IgM levels became moderate or low (Spearman’s ρ = 0.56 [p < 0.001] and 0.32 [p < 0.001], respectively) (Supplementary Fig. 1).

### The occurrence of adverse effects has no association with the BNT162b2-elicited neutralizing activity levels

**Figure 2:**
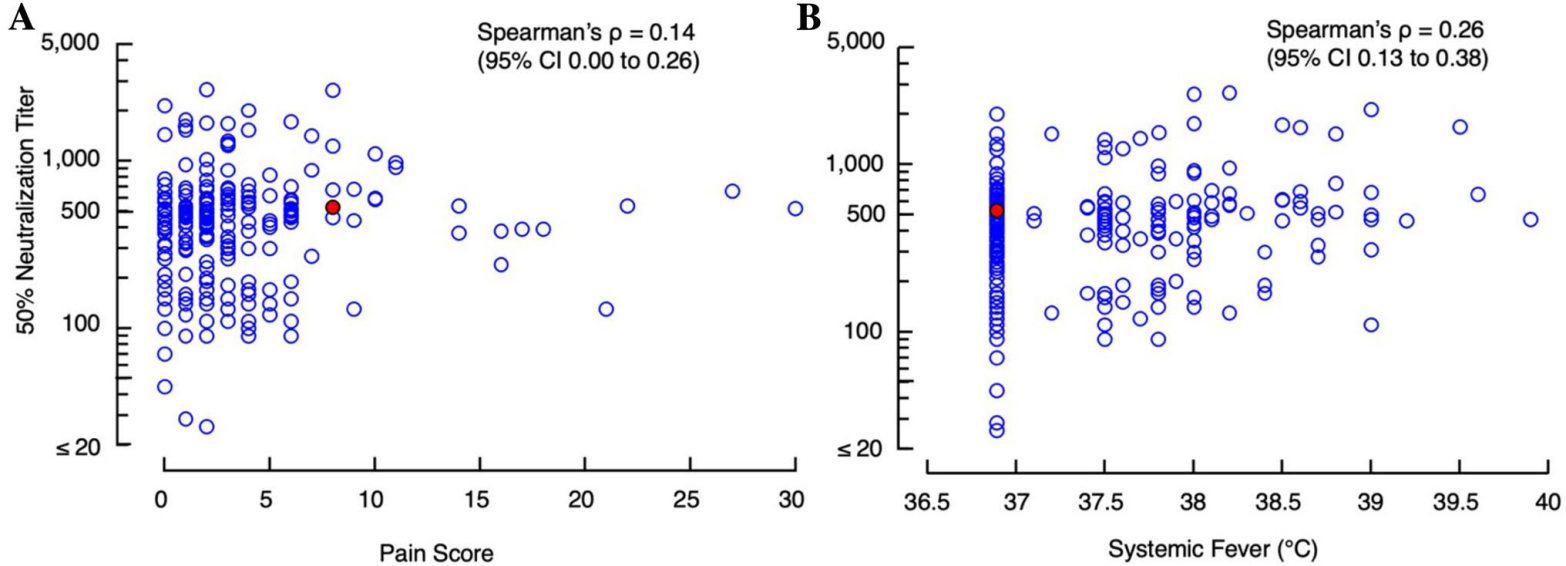
Correlations of neutralizing titers with injection-site pain scores and systemic fever grades. (**A**) No correlation was seen between NT_50_ values and injection-site pain (Spearman’s ρ = 0.14; 95% CI 0.00–0.26, *p* = 0.043). The injection-site pain following the 2nd BNT162b2 dose was scored by using the short-form McGill Pain Questionnaire^23^. (**B**) Correlation was negligible between NT_50_ values and systemic fever grades (Spearman’s ρ = 0.26; 95% CI 0.13–0.38, *p* < 0.001). A solid-red circle indicates a person with previous SARS-CoV-2 infection documented.

Commonly observed adverse events reported following BNT162b2 vaccination include injection-site pain, systemic fever, headache, and fatigue^11^. In the present study, the events were observed largely more often following the 2nd dose (Supplementary Fig. 2) as previously reported by Polack et al.^11^. Pains in the inject-site were reported by 67.6 and 61.6% of the participants and systemic fever (≥ 37.1℃) was reported by 3.6 and 46.4% of the participants following the 1st and 2nd doses, respectively. Fever was measured when each participant felt feverish at any time points over weeks following the 1st and 2nd vaccinations. Since the severity of pains can be more easily quantified than other adverse effects such as headache and fatigue, the possible correlate of the NT_50_ values with the severity of pains rated with the short form McGill pain questionnaire^23^ was first examined. No correlation was seen between the NT_50_ values and the pain grades assessed following the 2nd dose (Spearman’s ρ = 0.14; 95% CI 0.00–0.26, *p* = 0.043). The correlation was also negligible between the NT_50_ values and the incidence of systemic fever (Spearman’s ρ = 0.26; 95% CI 0.13–0.38, p < 0.001) (Fig. 2A, B).

### The average half-life of neutralizing activity in the vaccinees is approximately 67.8 days and the average time-length for their sera to lose the detectable activity is 198.3 days

**Figure 3:**
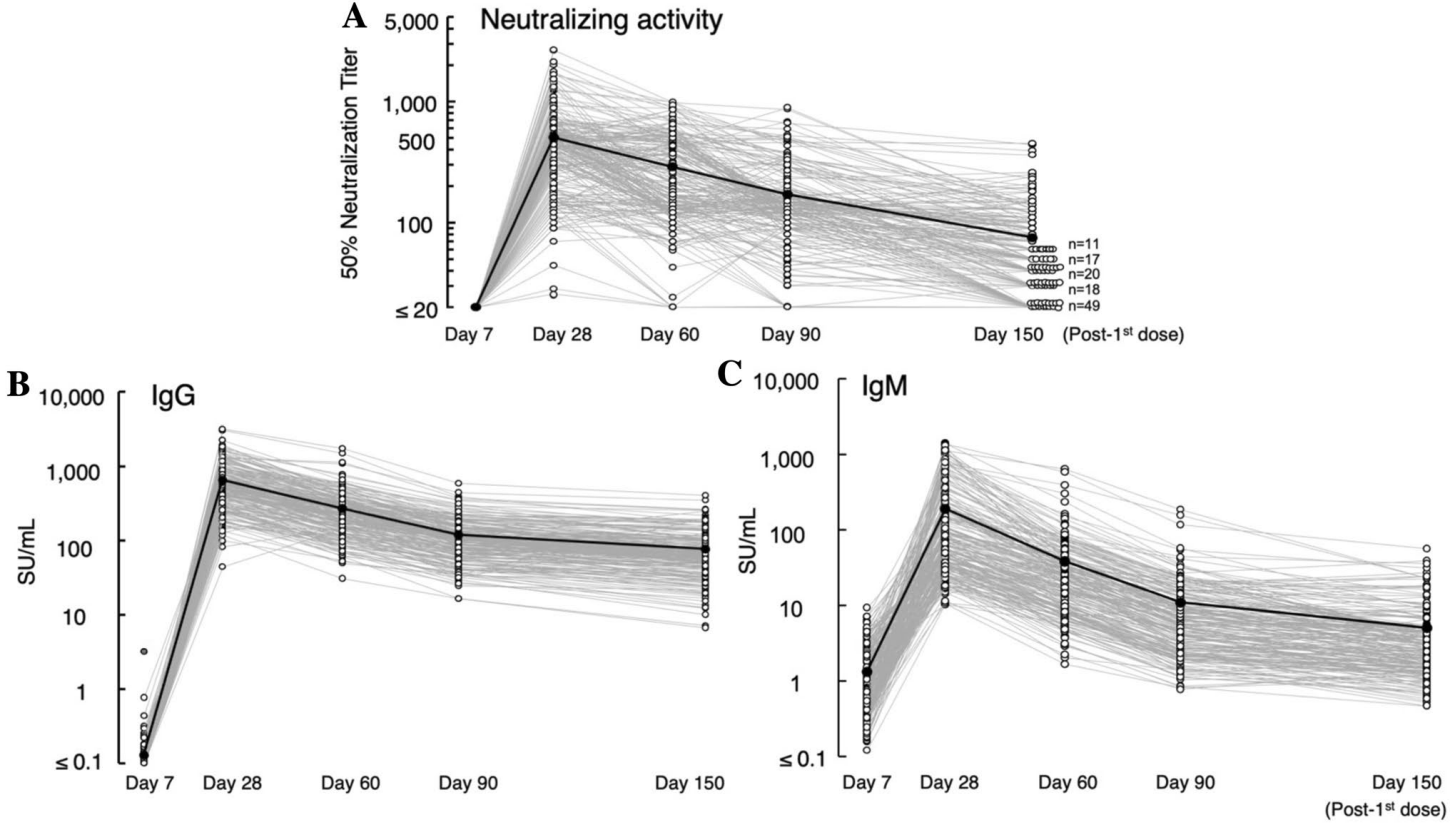
Kinetics of neutralizing activity and S1-binding-IgG and -IgM levels. Time-course analyses of neutralizing activity for 90 days were conducted. The 1st vaccine was administered on day 0, and the 2nd vaccine on day 21. Blood samples from vaccinated individuals were obtained on days 7, 28, 60, 90 and 150 post 1st dose as illustrated in Table 1. (**A**) Neutralizing activity is shown as NT_50_ (50% neutralizing titer). The NT_50_ value of 20 is the detection limit and values determined to be less than 20 were treated as 20. (**B**, **C**) Kinetics of S1-binding-IgG and -IgM levels are shown. The average values of each data point are shown in black solid circles, which are connected with solid black lines. One participant, who had been infected with SARS-CoV-2 with PCR-positivity documented, is indicated as a solid-red solitary circle in (**B**) and (**C**). This participant was excluded from all analyses at later timepoints.

Considering that recent multiple clinical studies strongly suggest that the presence of high-level neutralizing antibodies is generally sufficient to confer protection against SARS-CoV-2 infection and that the protection against COVID-19 development is largely explained by robust SARS-CoV-2-neutralizing antibody response^9–11^. If so, the once-established neutralizing antibody levels will decrease in time. We thus examined at what rate the levels of NT_50_ and S1-bindng-IgG and -IgM levels change by determining those levels from the data on day 28 (n = 220), day 60 (n = 211), and day 90 (n = 210) post-1st dose (Fig. 3A–C). The reduction of all NT_50_, IgG, and IgM levels from day 28 through day 90 post-1st dose was found to occur virtually linearly. By calculation, the predicted average half-life of all the NT_50_ values turned out to be 67.8 days and those of S1-binding-IgG and IgM levels were 53.5 days and 43.6 days, respectively (Fig. 3A–C). The half-life of the NT_50_ values and that of S1-binding-IgG were reasonably comparable, corroborating that the neutralizing activity largely resides in the S1-binding IgG fraction. As seen, chronologically linear nature of the reduction was identified between day 28 and 90 for both neutralization and antibody titers. However, the rate of decline was found to be more modest and slower between the data on day 90 and day 150 post-1st dose (especially in antibody titers) (Fig. 3A–C). We also asked whether the chronological decay rate of neutralization titers and S1-binding-IgG and -IgM differs among three age subgroups: (1) 20–39 yo, (2) 40–59 yo, and (3) 60 s and beyond. No significant difference was identified among the three age subgroups in the levels of neutralizing titers, IgG, or IgM levels (*p* = 0.60, 0.16, and 0.11, respectively: Supplementary Fig. 3A–C). The present data suggest that vaccinated individuals with good neutralization response would lose BNT162b2’s protection in 6–7 months without regard to age subgroups unless such people achieve robust immune boost response upon the future exposure to SARS-CoV-2. Otherwise, they should be protected by another booster vaccine dose or by other protective means.

### Neutralization titers, S1-binding-IgG levels, and pain scores in the injection site were greater in women than in men

We then asked whether there were differences between genders in neutralization activity levels, S1-bindng-IgG and -IgM levels, injection-site pain scores, and systemic fever grades. Statistically significant differences were identified in the levels of neutralization determined on 60 and 90 days post-1st dose (*p* = 0.002 and 0.002, respectively), S1-binding-IgG levels determined on 28, 60, and 90 days (*p* < 0.001, p = 0.001, and *p* =  < 0.001, respectively) post-1st-dose, and S1-binding-IgM levels on 60 and 90 days post-1st dose (*p* = 0.025 and 0.044, respectively) (Supplementary Fig. 4A–C). The injection-site pain score was greater in women (*p* < 0.001) (Supplementary Fig. 4D). However, there was no difference in systemic fever grades between genders (Supplementary Fig. 4E).

### Some sera retain potent activity against various VOCs, but others showed substantially less potent or undetectable activity

**Figure 4:**
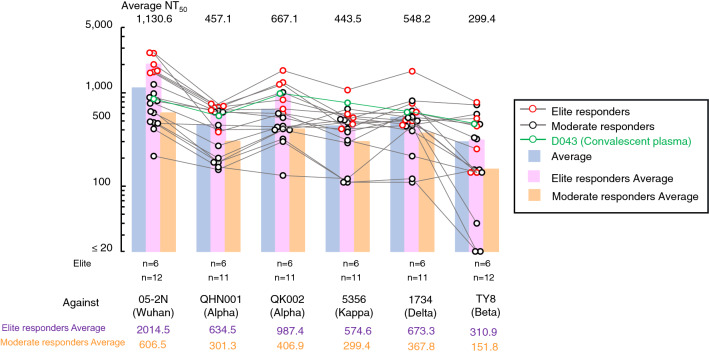
Blockade of the infectivity and replication of SARS-CoV-2 variants by vaccinees’ sera on day 28 post-1st dose. The activity of vaccinees’ sera to block the infectivity and replication of 5 SARS-CoV-2 variants (alpha variants: SARS-CoV-2_QHN001_ and SARS-CoV-2_QK002_; a beta strain: SARS-CoV-2_TY8-612_; a delta strain: SARS-CoV-2_1734_; and a kappa strain: SARS-CoV-2_5356_) was evaluated. A Wuhan strain SARS-CoV-2_05-2 N_^41^ was employed as a reference SARS-CoV-2. Six sera were from elite responders (NT_50_ > 1,500) and 12 sera were from randomly-selected moderate responders (NT_50_ = 200 ~ 1,500). The NT_50_ titers of each sera against 6 SARS-CoV-2 strains are shown in red circles (for 6 elite responders) and in black circles (for 12 moderate responders). D043 is a sera from a COVID-19-convalescent patient^43^ and served as an internal control in the assays. *P* values for the difference between the averages of elite and moderate responders in each variant: < 0.001 (05-2 N), 0.006 (QHN001), 0.004 (QK002), 0.035 (5356), 0.119 (1734), and 0.371(TY8).

We finally asked whether the neutralizing antibodies elicited by BNT162b2 vaccination blocked the infectivity and replication of various variants of concerns (VOCs). To this end, we employed sera samples from 6 elite responders (NT_50_ values > 1,500: the top 4% of all participants’ NT_50_ values as determined on 28 days post-1st dose) and sera samples from twelve randomly-selected moderate responders (NT_50_ values = 200 ~ 1,500) and tested them for their inhibition of the infectivity and cytopathic effect of each variant in the VeroE6_TMPRSS2_ cell-based assay^24^. As shown in Fig. 4, sera from the elite responders (n = 6) showed potent inhibition against SARS-CoV-2_05-2 N_ (Wuhan strain, PANGO lineage B), while they showed less activity against SARS-CoV-2_QHN001_ and SARS-CoV-2_QK002_ (alpha), SARS-CoV-2_5356_ (kappa), SARS-CoV-2_1734_ (delta), and SARS-CoV-2_TY8-612_ (beta). Sera from moderate responders (n = 12) exerted less activity against the Wuhan strain than those from the elite responders. Some sera from the moderate responders also showed substantially low potency to all the VOCs tested. Notably, three sera from the moderate responders showed only marginal activity against SARS-CoV-2_TY8-612_ (beta). Two of those three samples had no detectable inhibitory activity against SARS-CoV-2_TY8-612_ (Fig. 4).

## Discussion

In this prospective observational study, 225 healthy individuals [physicians (n = 36), nurses (n = 125), and other healthcare professionals (n = 64)], who received two doses of 30 µg BNT162b2 (Pfizer–BioNTech) vaccine in February 2021, were enrolled, and the correlates of neutralization activity represented by 50% neutralization titers (NT_50_) determined by employing the target living VeroE6_TMPRSS2_ cells and live SARS-CoV-2 with ages, adverse effects (AEs) that occur often such as injection-site pain and systemic fever were examined. The kinetics of NT_50_ values and S1-binding antibody levels were also examined. There was a significant rise in the NT_50_ values as determined on day 28 post-1st dose (a week after post 2nd dose) compared to those on day 7 post-1st dose. Correlation was negligible between NT_50_ values and ages or systemic fever grades. In this regard, most adverse effects that occur within 1–3 days following vaccine doses are thought to be caused by the release of certain pyrogenic and inflammatory cytokines (*e.g.*, interleukin-1, interleukin-6, and tumor-necrosis factor) from antigen-presenting cells (APCs) such as macrophages and dendritic cells when they ingest and process the exogenous antigens (*i.e.*, SARS-CoV-2 spike antigens) and transmit the antigenic information to relevant immune cells. Such early-phase defense events include response to antigenic determinants irrelevant to neutralizing activity but those eliciting S1-binding antibodies. The release of the pyrogenic and inflammatory cytokines mostly subsides within days following the vaccine dose. The released cytokines activate the antigen-specific-antibody-producing B-cells, which respond to the processed antigenic determinants presented by the APCs and start to produce antigen-specific antibodies such as neutralizing antibodies as well as non-neutralizing but S1-binding antibodies. Such antigen-specifically-activated and antibody-producing B-cells continue to produce antibodies. In the case of BNT162b2 vaccination, it appears that it takes 10–12 days from the 1st vaccine dose for the vaccinated individuals to achieve the amounts of neutralizing antibodies that are enough to block the infection of substantial numbers of the virally-targeted cells and to inhibit further spread of the infection^10,11^. It is thought that the release of pyrogenic and inflammatory cytokines and the build-up of the protective antibody levels are different events, occurring chronologically ~ 10–12 days apart. These two different events appear to have resulted in the absence of significant correlates between NT_50_ levels and AEs examined in the present study.

In the present study, the NT_50_ values had a substantial correlation with S1-binding-IgG levels but had only moderate correlation with S1-binding IgM levels, suggesting that the major neutralizing activity resides within the S1-binding IgG fraction. Interestingly, the approximate half-life of NT_50_ values (69.7 days) and that of S1-binding-IgG levels (54.7 days) were reasonably close to each other, corroborating the assumption of the presence of the major fraction of neutralizing antibodies within IgG fraction. In human body, IgG has concentration-dependent half-life of approximately 21 days and IgM around 5–6 days^25^. By contrast, the half-lifes of NT_50_, S1-bindng-IgG, and -IgM levels determined in the present study were much longer with 39.0–54.7 days. This discrepancy is perhaps attributed to the persistence of continuously-antibody-producing B cells over weeks or months in the body of the participants following the vaccination^26,27^, thereby the half-lifes of neutralizing activity and S1-binding-IgG and -IgM levels have been extended as compared to the physiological half-lifes of IgG and IgM. In terms of the persistence for the efficacy of vaccine, Doria-Rose et al. reported that anti-SARS-CoV-2 antibodies persist through 6 months after the second dose of mRNA-1273 administration^28^. However, the study was of a relatively small scale (n = 33), and more definite data are needed for the understanding the nature of vaccine efficacy. In addition, Tartof et al. have also demonstrated that an examination of individuals receiving BNT-162b vaccine showed a decrease of protection, but the protection remained consistent up to 6 months^29^. Planas et al. have also showed that the sera from individuals had reduced at 16 weeks after receiving ChAdOx1 nCoV-19/AZD1222 vaccine, but such individuals with the reduced neutralizing activity had been well-protected from infection^30^. In our present study, we demonstrated that both neutralization and antibody titers decrease virtually linearly at the initial phase (up to ~ 3 months post-1st dose) but that reduction rate becomes rather slower when examined in ~ 5 months post-1st dose (Fig. 3A–C), which is in line with the data shown by others^29,30^. In addition, we examined N-IgG antibody levels (indicator for viral infection) in all participants at each time point (on Days 7, 28, 60, 90, and 150 post-1st dose) and found that none of the participants were positive for N-IgG throughout the test period. Moreover, none of the participants reported that they had no symptom suggesting new infection by SARS-CoV-2. Since the number of the participants is limited to 210 by day 150 post-1st dose, it is not clear as to whether the absence of infected participants over 150 days post-1st dose is due to the protective effect of the reduced neutralization and antibody levels.

In the present limited study enrolling only 225 participants, it is not possible to precisely associate the immune response seen with clinical outcomes such as development of severe COVID-19 and death. In this regard, Walsh et al. examined the immunogenicity of the Pfizer’s BNT162b2 vaccine and Polack et al. examined the outcomes in 21,728 vaccinated individuals and 21,720 placebo-receiving individuals^8,11^. In the two reports together, the BNT162b2 vaccine very effectively elicited good immune response; however, 5% of the participants in the study performed by Polack et al. contracted SARS-CoV-2 infection. In our present study, ~ 5% of the participants apparently failed to build reasonable neutralizing antibody levels (< × 110, on day 28; Fig. 3A). These probable low-responders might be susceptible to SARS-CoV-2 infection, might contract SARS-CoV-2 infection, and might develop severe COVID-19 and end up in death. However, as of this writing, reportedly none of the participants have contracted the virus or had COVID-19 symptom.

For the immunity against SARS-CoV-2, it is highly likely that the mucosal immunity also contributes the body’s defence against COVID-19. We have not examined the level of antibodies (especially IgA class) in mucosa after vaccinations in the present study, but that should be elucidated in future. It is also likely that the role and strength of mucosal immunity, antibody-dependent cellular cytotoxicity (ADCC), antibody-dependent cellular phagocytosis (ADCP), and such in the control of SARS-CoV-2 depends on the class of vaccines (mRNA-based vaccines, vector-based vaccines, or subunit vaccines) administered. The role of cellular immunity (mainly driven by cytotoxic T-cells and NK cells) after vaccination has also been reported upon anti-COVID-19 vaccination^31^. However, detailed dynamics of the potency and persistence of cellular immunity against SARS-CoV-2 still remain to be elucidated.

There is a growing body of evidence that COVID-19 results in more severe symptoms and greater mortality among men than among women^32,33^. A cohort study of 17 million adults in England has revealed a strong association between male sex and the risk of death from COVID-19 (hazard ratio 1.59, 95% confidence interval 1.53–1.65)^34^. In the present data set, significantly greater levels (p < 0.001) of NT_50_ (Days 60 and 90 post-1st dose), S1-binding-IgG (Days 28, 60 and 90), and -IgM (Days 60 and 90) were documented in women than in men when examined on 28, 60, and 90 days post-1st dose, while there was no difference in either of NT_50_, S1-binding-IgG or -IgM levels on day 7 post-1st dose (Supplementary Fig. 4A–C). These results apparently relate to the findings by others reporting that women, in general, have more robust ability to control infectious pathogens (*i.e.*, SARS-CoV-2) than men^34,35^. Indeed, there is increasing evidence indicating strong correlation between SARS-CoV-2-neuralizing antibody titers and clinical efficacy, suggesting that a neutralizing antibody response provides the primary contribution to protection against COVID-19^36^ and that the presence of high levels of neutralizing antibody is largely sufficient for protection against SARS-CoV-2 infection and clinical onset upon exposure to the virus^37,38^. In fact, Imai et al*.* have reported that the administration of convalescent plasma from previously-SARS-CoV-2-infected hamsters completely protected newly SARS-CoV-2-exposed hamsters from contracting viral pneumonitis^39^. Thus, the greater neutralizing activity in women than in men observed in the present study can contribute at least in part to the gender differences in COVID-19 disease outcomes.

We also examined how the BNT162b2-elicited neutralizing antibodies blocked the infectivity and cytopathic effect of five different variants of concerns in the cell-based assays using various infectious variants (one Wuhan strain, two alpha strains, one strain each of beta, delta and kappa strains). Six selected sera from elite responders showed quite potent activity to the alpha, kappa, and delta variants, while they exerted relatively moderate activity against the beta strain (Fig. 4). On the other hand, one of the randomly-selected 12 sera from moderate responders showed a marginal activity (NT_50_ value of 40) and two of them failed to show detectable activity (NT_50_ values < 20) against the beta strain (Fig. 4). These data suggest that BNT162b2-receiving vaccinees who develop high magnitudes of neutralizing antibody should probably be well protected against the infection by most variants; however, those who develop only low levels of neutralizing antibody may be vulnerable to the infection by certain variants such as beta strains. If so, to such low-responders to BNT162b2 even after the 2nd dose, an additional 3rd dose may be needed. If the 3rd dose of the same vaccine fails to elicit good levels of neutralizing antibodies, new types of vaccines with different platform have to be stratified.

## Methods

### Participants and sera specimens

Sera were collected from 225 vaccinated (BNT-162b, 30 μg/dose) health care workers at JCHO Kumamoto General Hospital (Kumamoto, Japan). All the 225 participants were of Japanese citizen. Sera samples were analyzed at the National Center for Global Health and Medicine (NCGM) in Tokyo. The Ethics Committees from the Kumamoto General Hospital and NCGM approved this study (Kumamoto General Hospital No. 180, and NCGM-G-004176–00, respectively). Each participant provided a written informed consent, and this study abided by the Declaration of Helsinki principles. The vaccination (on days 0 and 21) and sera collection (from day 7 through day 90 post-1st dose) were conducted as shown in Table 1.

### Cells and viruses

VeroE6_TMPRSS2_ cells^24^ were obtained from Japanese Collection of Research Bioresources (JCRB) Cell Bank (Osaka, Japan). VeroE6_TMPRSS2_ cells were maintained in DMEM supplemented with 10% FCS, 100 µg/ml of penicillin, 100 µg/ml of streptomycin, and 1 mg/mL of G418. SARS-CoV-2 NCGM-05-2 N strain (SARS-CoV-2_05-2 N_) was isolated from nasopharyngeal swabs of a patient with COVID-19, who was admitted to the NCGM hospital^40,41^. Five clinically isolated SARS-CoV-2 mutant strains were used in the current study: two B.1.1.7 (alpha) strains [hCoV-19/Japan/QHN001/2020 (SARS-CoV-2_QHN001_, GISAID Accession ID; EPI_ISL_804007) and hCoV-19/Japan/QK002/2020 (SARS-CoV-2_QK002_, GISAID Accession ID; EPI_ISL_768526)] and a B.1.351 (beta) strain [hCoV-19/Japan/TY8-612-P0/2021 (SARS-CoV-2_TY8-612_)] were obtained from National Institute of Infectious Diseases, Tokyo, Japan. A B.1.617.1 (kappa) strain [TKYTK5356_2021 (SARS-CoV-2_5356_, DDBJ Accession ID; LC633761)] and a B.1.617.2 (delta) strain [hCoV-19/Japan/TKYK01734/2021 (SARS-CoV-2_1734_, GISAID Accession ID; EPI_ISL_2080609)] were provided from Tokyo Metropolitan Institute of public Health, Tokyo, Japan. Each variant was confirmed to contain each VOC-specific amino acid substitutions before the assays conducted in the present study (vide infra).

### Neutralization assay

The neutralizing activity of sera on days 7, 28, 60, 90, and 150 from vaccinated individuals was determined by quantifying the sera-mediated suppression of the cytopathic effect (CPE) of each SARS-CoV-2 strain in VeroE6_TMPRSS2_ cells as previously described with minor modifications^40^. In brief, each sera was fourfold serially diluted in culture medium. The diluted sera were incubated with 100 50% tissue culture infectious dose (TCID_50_) of viruses at 37 °C for 20 min (final sera dilution range of 1:20–1:4000), after which the sera-virus mixtures were inoculated to VeroE6_TMPRSS2_ cells (1.0 × 10^4^/well) in 96-well plates. For SARS-CoV-2 strains used in this assay are as follows: a wild-type strain, SARS-CoV-2_05-2 N_ (PANGO lineage B)^40,41^, two alpha variants (SARS-CoV-2_QHN001_ and SARS-CoV-2_QK002_), a beta variant SARS-CoV-2_TY8-612_, a delta variant SARS-CoV-2_1734_, and a kappa variant SARS-CoV-2_5356_. After culturing the cells for 3 days, the levels of CPE observed in SARS-CoV-2-exposed cells were determined using the WST-8 assay employing Cell Counting Kit-8 (Dojindo, Kumamoto, Japan). The sera dilution that gave 50% inhibition of CPE was defined as the 50% neutralization titer (NT_50_). Each sera was tested in duplicate.

### Measurement of anti-SARS-CoV-2 antibody titers

Measurement of 3 anti-SARS-CoV-2 antibody levels (anti-S1-IgG, anti-S1-IgM, and anti-N-IgG) in sera of each participant obtained on days 7, 28, 60, 90, and 150 from 1st dose was performed using the chemiluminescence enzyme immunoassay (CLEIA) platform (HISCL) manufactured by Sysmex Co. (Kobe, Japan) as previously reported^22^.

### Statistical analyses

Out of the 225 participants, one participant, who had been infected by SARS-CoV-2 with PCR positivity documented was primarily excluded. Demographic characteristics of the participants are described in Table 1. Correlates of neutralizing activity levels with S1-binding-IgG and -IgM levels, ages, pain scores in the injection-site, and systemic fever up to 40℃ were examined by Spearman rank correlation coefficient. Also, neutralizing activity levels, S1-binding-IgG, and -IgM, pain scores, systemic fever were compared between genders using the Wilcoxon rank sum test. As for the normal body temperature of the participants, 36.89 degree was used, which has been reported to be a normal body temperature in Japanese^42^. Percentage of the adverse events reported in writing following the 1st and 2nd dose administration were determined and assessed in regard to gender. The differences in neutralization activity between each measurement time point were tested by the Wilcoxon rank sum test, and were assessed among categorized age subgroups. Similarly, difference of S1-binding-IgG and -IgM levels among time points were tested. We considered the computed correlation coefficient low if the value was below 0.4, moderate if the value was between 0.4 and 0.7 and high if the value was above 0.7 according to Guilford’s Rule of Thumb. The threshold for statistical significance was *p* < 0.05. All the analyses were conducted with the use of R, version 4.1.0 (R Foundation for Statistical Computing).

## Supplementary Information

Supplementary Information.

## References

[1] World Health Organization. Coronavirus disease (COVID-19) outbreak situation. https://www.who.int/publications/m/item/weekly-epidemiological-update-on-covid-19---29-june-2021 (2021). 37184163

[2] Wu F (2020). A new coronavirus associated with human respiratory disease in China. Nature.

[3] Zhou P (2020). A pneumonia outbreak associated with a new coronavirus of probable bat origin. Nature.

[4] Mitsuya H, Kokudo N (2020). Sustaining containment of COVID-19: Global sharing for pandemic response. Glob. Health Med..

[5] Dal-Re R (2021). Ongoing and future COVID-19 vaccine clinical trials: Challenges and opportunities. Lancet Infect. Dis..

[6] Cohen J (2020). Effective vaccine offers shot of hope for pandemic. Science.

[7] Richman DD (2021). COVID-19 vaccines: Implementation, limitations and opportunities. Glob. Health Med..

[8] Walsh EE (2020). Safety and immunogenicity of two RNA-based Covid-19 vaccine candidates. N. Engl. J. Med..

[9] Pfizer. News/Pfizer and BioNTech conclude phase 3 study of COVID-19 vaccine candidate, meeting all primary efficacy endpoints. https://www.pfizer.com/news/press-release/press-release-detail/pfizer-and-biontech-conclude-phase-3-study-covid-19-vaccine (2021).

[10] Baden LR (2021). Efficacy and safety of the mRNA-1273 SARS-CoV-2 vaccine. N. Engl. J. Med..

[11] Polack FP (2020). Safety and efficacy of the BNT162b2 mRNA Covid-19 vaccine. N. Engl. J. Med..

[12] Voysey M (2021). Safety and efficacy of the ChAdOx1 nCoV-19 vaccine (AZD1222) against SARS-CoV-2: An interim analysis of four randomised controlled trials in Brazil, South Africa, and the UK. Lancet.

[13] Johnson & Johnson. Johnson & Johnson COVID-19 vaccine authorized by U.S. FDA for emergency use—First single-shot vaccine in fight against global pandemic. https://www.jnj.com/johnson-johnson-covid-19-vaccine-authorized-by-u-s-fda-for-emergency-usefirst-single-shot-vaccine-in-fight-against-global-pandemic (2021).

[14] Sadoff J (2021). Safety and efficacy of single-dose Ad26.COV2.S vaccine against Covid-19. N. Engl. J. Med..

[15] Korber B (2020). Tracking changes in SARS-CoV-2 spike: Evidence that D614G increases infectivity of the COVID-19 virus. Cell.

[16] Xie X (2021). Neutralization of SARS-CoV-2 spike 69/70 deletion, E484K and N501Y variants by BNT162b2 vaccine-elicited sera. Nat. Med..

[17] Cele S (2021). Escape of SARS-CoV-2 501Y.V2 from neutralization by convalescent plasma. Nature.

[18] Heath PT (2021). Safety and efficacy of NVX-CoV2373 Covid-19 vaccine. N. Engl. J. Med..

[19] Novavax. Novavax COVID-19 vaccine demonstrates 89.3% efficacy in UK phase 3 Trial. https://www.globenewswire.com/news-release/2021/01/28/2166253/0/en/Novavax-COVID-19-Vaccine-Demonstrates-89-3-Efficacy-in-UK-Phase-3-Trial.html (2021).

[20] Shinde V (2021). Efficacy of NVX-CoV2373 Covid-19 vaccine against the B.1.351 variant. N. Engl. J. Med..

[21] Wall EC (2021). Neutralising antibody activity against SARS-CoV-2 VOCs B.1.617.2 and B.1.351 by BNT162b2 vaccination. Lancet.

[22] Noda K (2021). A novel highly quantitative and reproducible assay for the detection of anti-SARS-CoV-2 IgG and IgM antibodies. Sci. Rep..

[23] Melzack R (1987). The short-form McGill Pain Questionnaire. Pain.

[24] Matsuyama S (2020). Enhanced isolation of SARS-CoV-2 by TMPRSS2-expressing cells. Proc. Natl. Acad. Sci. U S A.

[25] Waldmann TA, Strober W (1969). Metabolism of immunoglobulins. Prog Allergy.

[26] Turner JS (2021). SARS-CoV-2 mRNA vaccines induce persistent human germinal centre responses. Nature.

[27] Stephens DS, McElrath MJ (2020). COVID-19 and the path to immunity. JAMA.

[28] Doria-Rose N (2021). Antibody persistence through 6 months after the second dose of mRNA-1273 vaccine for Covid-19. N. Engl. J. Med..

[29] Tartof SY (2021). Effectiveness of mRNA BNT162b2 COVID-19 vaccine up to 6 months in a large integrated health system in the USA: A retrospective cohort study. Lancet.

[30] Planas D (2021). Reduced sensitivity of SARS-CoV-2 variant Delta to antibody neutralization. Nature.

[31] Oberhardt V (2021). Rapid and stable mobilization of CD8(+) T cells by SARS-CoV-2 mRNA vaccine. Nature.

[32] Chen N (2020). Epidemiological and clinical characteristics of 99 cases of 2019 novel coronavirus pneumonia in Wuhan, China: A descriptive study. Lancet.

[33] Yang X (2020). Clinical course and outcomes of critically ill patients with SARS-CoV-2 pneumonia in Wuhan, China: A single-centered, retrospective, observational study. Lancet Respir. Med..

[34] Williamson EJ (2020). Factors associated with COVID-19-related death using OpenSAFELY. Nature.

[35] Collazos J, Asensi V, Carton JA (2007). Sex differences in the clinical, immunological and virological parameters of HIV-infected patients treated with HAART. AIDS.

[36] Voysey M (2021). Single-dose administration and the influence of the timing of the booster dose on immunogenicity and efficacy of ChAdOx1 nCoV-19 (AZD1222) vaccine: A pooled analysis of four randomised trials. Lancet.

[37] Khoury, D., *et al.* What level of neutralising antibody protects from COVID-19? *medRxiv* (2021).

[38] Earle, K. A., *et al.* Evidence for antibody as a protective correlate for COVID-19 vaccines. *medRxiv* (2021). 10.1016/j.vaccine.2021.05.063 PMC8142841 34210573

[39] Imai M (2020). Syrian hamsters as a small animal model for SARS-CoV-2 infection and countermeasure development. Proc. Natl. Acad. Sci. U S A.

[40] Maeda K (2021). Neutralization of SARS-CoV-2 with IgG from COVID-19-convalescent plasma. Sci. Rep..

[41] Hattori SI (2020). GRL-0920, an indole chloropyridinyl ester, completely blocks SARS-CoV-2 infection. MBio.

[42] Wada Y (2009). For health care by taking the body temperature. Jpn. Soc. Mech. Eng..

[43] Takamatsu, Y., *et al.* Highly-neutralizing COVID-19-convalescent-plasmas potently block SARS-CoV-2 replication and pneumonia in Syrian hamsters. *bioRxiv* (2021). 10.1128/jvi.01551-21 PMC8865546 34818068

