# Supplementary material for: Correlates of neutralizing/SARS-CoV-2-S1-binding antibody response with adverse effects and immune kinetics in BNT162b2-vaccinated individuals
Source: Sci Rep. 2021 Nov 24;11:22848. doi: 10.1038/s41598-021-01930-y (PMC8613264; doi:10.1038/s41598-021-01930-y)
Supplement: Supplementary file 1 — Supplementary Information. [file 41598_2021_1930_MOESM1_ESM.pdf]

Supplementary Figures

Supplement to: Maeda K, Amano M, Uemura Y et al. Correlates of Neutralizing/SARS-CoV-2-S1-binding Antibody Response with Adverse Effects and Immune Kinetics in BNT162b2-Vaccinated Individuals.

Contents

Supplemental figures 1 to 4

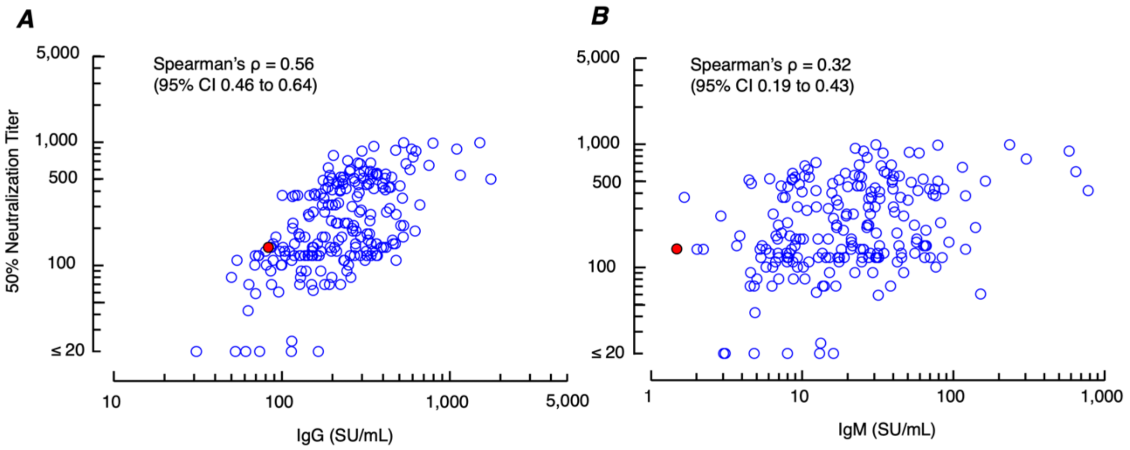

Supplementary Figure 1. A. Correlation between neutralizing activity and IgG levels on day 60 post 1st dose. Moderate correlation is identified (Spearman's ρ=0.56, p<0.001; 95% CI 0.46 to 0.64) between NT_{50} values and S1-binding-IgG levels in samples obtained on day 60 post-1st dose. B. Correlation between neutralizing activity and S1-binding-IgM levels on day 60 post-1st dose. Low correlation is seen between neutralizing titers and S1-binding-IgM levels (Spearman's ρ=0.32, p<0.001; 95% CI 0.19 to 0.43). A red-solid circle denotes a person with previous SARS-CoV-2 infection documented.

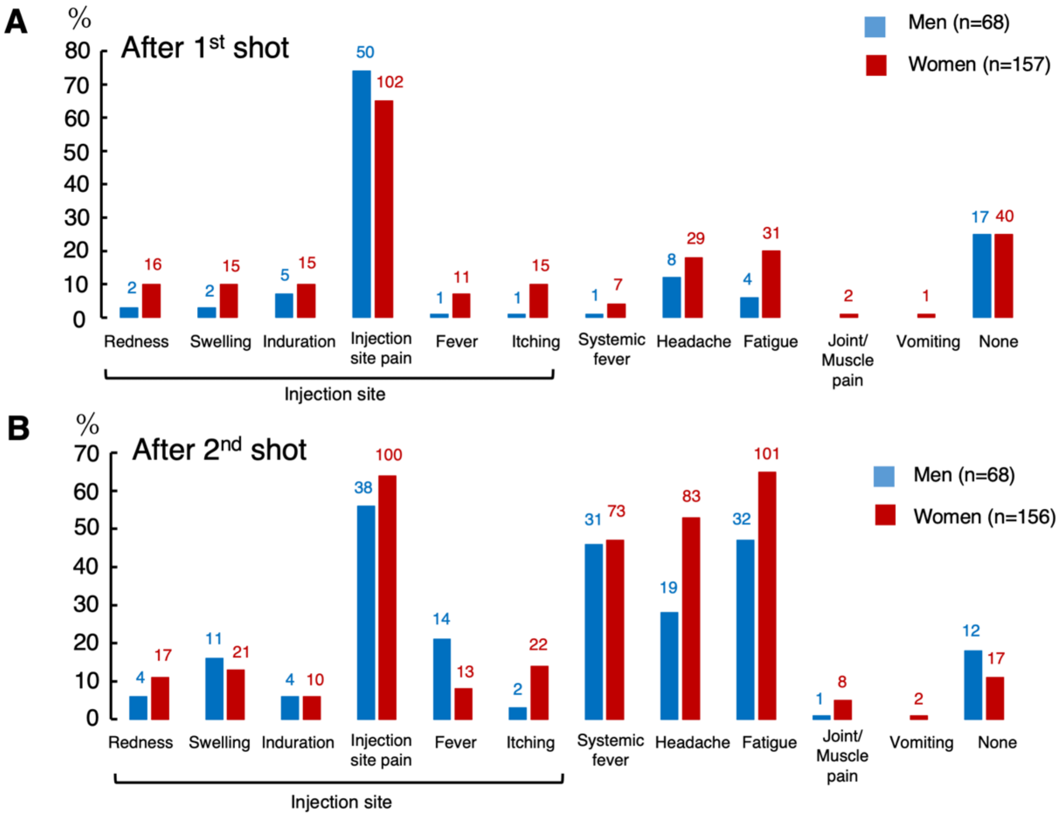

Supplementary Figure 2. Incidences of adverse effects reported after the 1st vaccination (A) and the 2nd vaccination (B). A total of 225 (Men: 68, Women: 157) participants reported after the 1st dose, and a total of 224 (Men: 68, Women: 156) participants after the 2nd dose. Systemic fever of ≥37.1℃ and pain scores of ≥1 were taken into the analyses. The number at the top of each bar denotes the number of individuals reporting each AE.

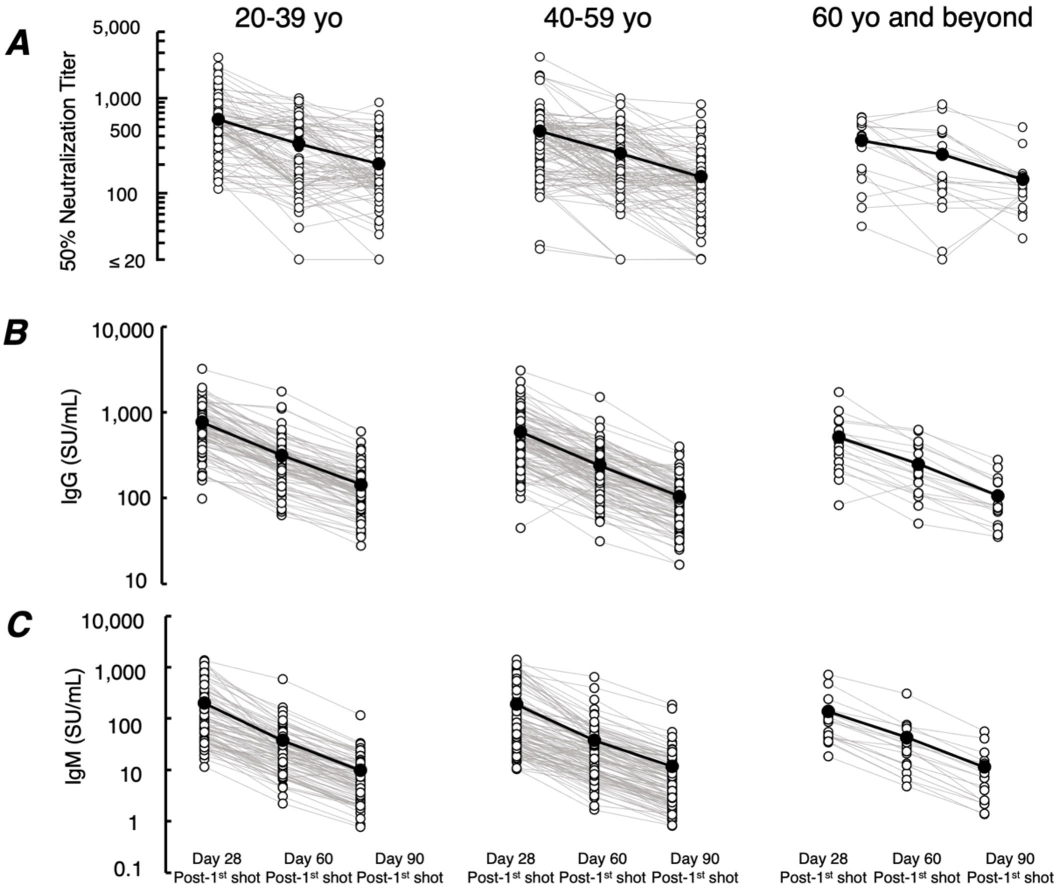

Supplementary Figure 3. Decline of neutralization titers and S1-binding antibody levels in three age groups. The chronological decline rates of neutralization titers (A), S1-binding-IgG (B), and -IgM levels (C) among three age groups; (i) 20-39 yo, (ii) 40-59 yo, and (iii) 60's and beyond, were evaluated. No significant difference was identified among the three age groups in the decline rates of neutralizing titers, IgG, or IgM levels (p=0.596, 0.163, and 0.106, respectively). Statistical analysis was conducted with the mixed-effects model including time, age category and time-age category interaction term and intercept as a random effect. A solitary open circle on day 28 in the 20-39 yo subgroup illustrated in B denotes a participant who did not provide blood sample for unknown reason on days 60 and 90 post-1st dose.

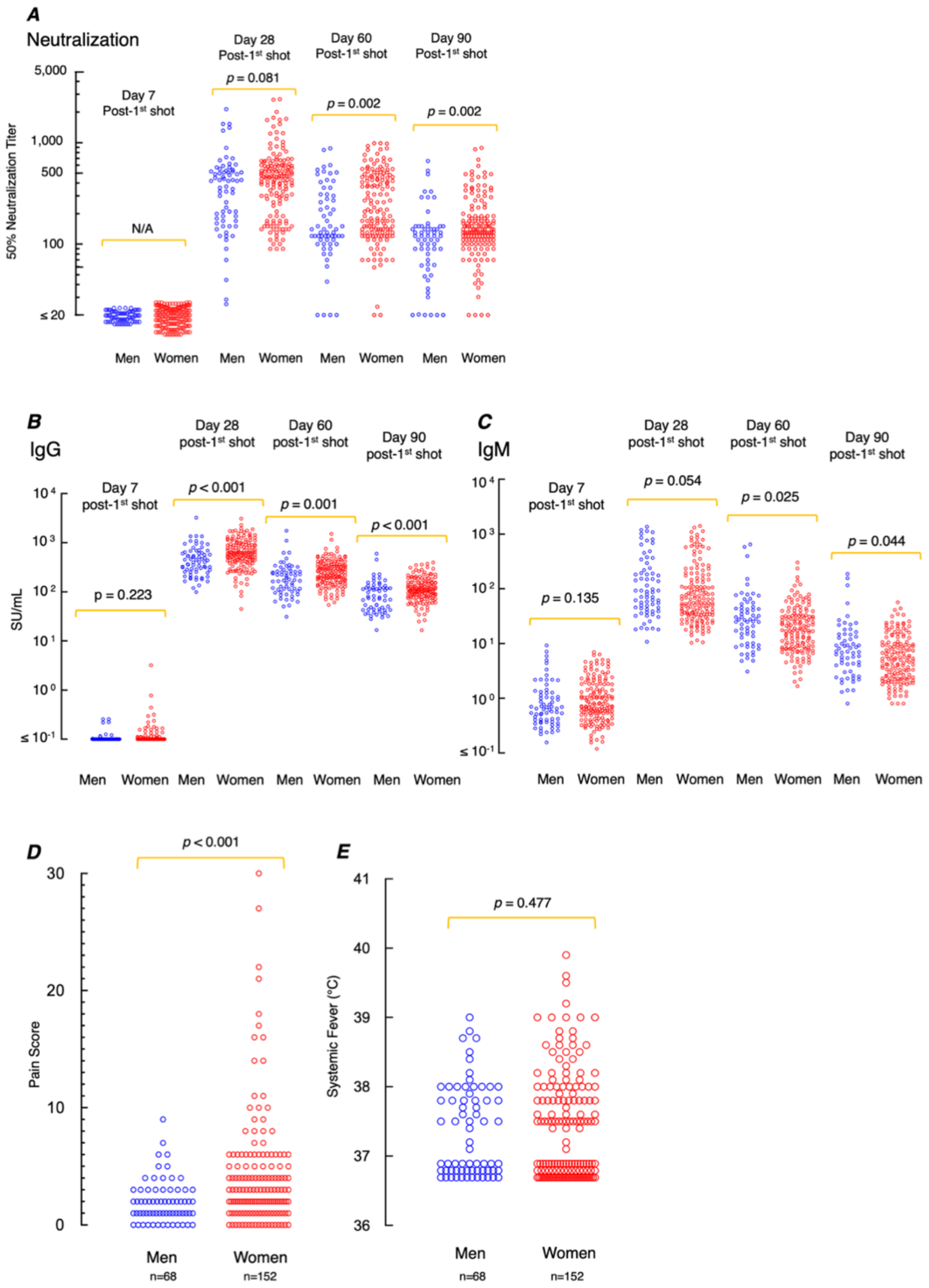

Supplementary Figure 4. Neutralization activity, S1-binding-IgG and -IgM levels, and pain scores are greater in women than in men. A. Neutralization activity was greater in women than in men at two timepoints (days 60 and 90 post-1st dose). B and C. S1-binding-IgG levels were greater in women at all three timepoints (days 28, 60, and 90 post-1st dose) and S1-binding-IgM levels greater in women at two timepoints (day 60 and 90 post-1st dose). D, E. Injection-site pain scores and systemic fever grades in men and women. Scores of injection-site pain were greater in women than in men (p<0.001)(D), while no difference was seen in systemic fever grades between the two groups (p=0.477)(E). Statistical significance was evaluated using Wilcoxon rank sum test. N.A., not applicable.
